# High-density lipoprotein subclass and particle size in coronary heart disease patients with or without diabetes

**DOI:** 10.1186/1476-511X-11-54

**Published:** 2012-05-15

**Authors:** Li Tian, Shiyin Long, Chuanwei Li, Yinghui Liu, Yucheng Chen, Zhi Zeng, Mingde Fu

**Affiliations:** 1 Laboratory of Endocrinology and Metabolism, West China Hospital, Sichuan University, Chengdu, Sichuan, 610041, People’s Republic of China; 2 State Key Laboratory of Biotherapy, Sichuan University, New building 6, #16 Section 3, People South Road, Chengdu, Sichuan, 610041, People’s Republic of China; 3 Department of Biochemistry and Molecular Biology, University of South China, Hengyang, Hunan, People’s Republic of China; 4 Cardiovascular department of West China Hospital, Sichuan University, Chengdu, Sichuan, 610041, People’s Republic of China

**Keywords:** Coronary heart disease, High density lipoprotein subclasses, Diabetic mellitus, Fasting plasma glucose

## Abstract

**Background:**

A higher prevalence of coronary heart disease (CHD) in people with diabetes. We investigated the high-density lipoprotein (HDL) subclass profiles and alterations of particle size in CHD patients with diabetes or without diabetes.

**Methods:**

Plasma HDL subclasses were quantified in CHD by 1-dimensional gel electrophoresis coupled with immunodetection.

**Results:**

Although the particle size of HDL tend to small, the mean levels of low density lipoprotein cholesterol(LDL-C) and total cholesterol (TC) have achieved normal or desirable for CHD patients with or without diabetes who administered statins therapy. Fasting plasma glucose (FPG), triglyceride (TG), TC, LDL-C concentrations, and HDL_3_ (HDL_3b_ and _3a_) contents along with Gensini Score were significantly higher; but those of HDL-C, HDL_2b+preβ2_, and HDL_2a_ were significantly lower in CHD patients with diabetes versus CHD patients without diabetes; The preβ_1_-HDL contents did not differ significantly between these groups. Multivariate regression analysis revealed that Gensini Score was significantly and independently predicted by HDL_2a_, and HDL_2b+preβ2_.

**Conclusions:**

The abnormality of HDL subpopulations distribution and particle size may contribute to CHD risk in diabetes patients. The HDL subclasses distribution may help in severity of coronary artery and risk stratification, especially in CHD patients with therapeutic LDL, TG and HDL levels.

## Introduction

The diabetes is a major contributor to coronary artery disease (CAD) morbidity and mortality. It has been reported that adults with diabetes are at a 2- to 4-fold increased risk of CAD events relative to those without DM [1-5] and are at about 60% increased risk of early mortality [6]. Features of the well documented “atherogenic lipid pattern”, characterized by lowered high-density lipoprotein cholesterol (HDL-C), and elevated serum triglycerides (TG) [7], are presented in diabetic individuals. On the other hand, it is well established that plasma concentrations of HDL-C are inversely correlated with the risk of atherosclerosis and cardiovascular disease (CVD) [8].

HDL particles are heterogeneous in size and composition. Using 1-dimensional gel electrophoresis and immunoblotting, HDL can be subdivided into preβ_1_-HDL, HDL_3c_, HDL_3b_, and HDL_3a_ as well as HDL_2a_ and HDL_2b+preβ2-HDL_. Despite the substantial epidemiological data suggesting a cardioprotective role for HDL, much remains unknown about the anti-atherothrombogenic properties of different particles that comprise this class of lipoproteins [9]. CHD patients often have increased small discoidal HDL particles and decreased large α-1 and α-2 HDL particles [10]. The preβ_1_-HDL was identified as a novel and independent predictor of myocardial infarction (MI) above and beyond traditional CHD risk factors [11]. Other study showed that small HDL particle size was associated with an increased CHD risk, but this association was largely explained by traditional risk factors [12]. The progressive insulin resistance(IR) was associated a decrease in HDL size as a result of depletion of large HDL_2a_ and HDL_2b_ particles and a modest increase in small HDL. The HDL subclass pattern is consistent with impaired reverse cholesterol transport (RCT) in IR individuals [13]. Considering that high-risk individuals are often treated with statins, development of diagnostic and treatment strategies to study and target HDL metabolism must involve not only the absolute concentration of HDL-C, but also the functional properties of HDL particles [14]. Which might provide more useful information for risk stratification in potentially high-risk individuals and particularly in those patients treated with lipid-altering therapy. In this study, we examined the HDL subclasses distribution profile in CHD patients with or without diabetes by one-dimensional gradient gel electrophoresis.

### Subjects and methods

#### Subjects

Two hundred and twelve patients with established CHD participated in this study (These patients who were hospitalized in cardiovascular department of West China Hospital, Sichuan University). Quantitative coronary angiography (QCA) was performed at the Cardiology Department of the West China Hospital, Sichuan University. Diagnosis was based on clinical history and was confirmed by QCA. The results of angiographic examination were regarded positive for coronary atherosclerosis only if one or more major coronary arteries (right coronary, left main coronary, left anterior descending, and circumflex) had at least 50% stenosis of the luminal area. All angiograms were evaluated by two experienced physicians blinded to the study, and the severity of coronary artery disease was assessed by using the Gensini score system [15] which grades narrowing of the lumens of the coronary arteries as: 1 for 1–25% narrowing, 2 for 26–50% narrowing, 4 for 51–75%, 8 for 76–90%, 16 for 91–99% and 32 for a completely occluded artery. This score is then multiplied by a factor according to the importance of the coronary artery as follows: 5 for a left main stem (LMS) lesion, 2.5 for proximal left anterior descending artery (LAD) and proximal circumflex artery (CX) lesions, 1.5 for a mid-LAD lesion, and 1 for distal LAD, mid/distal CX and right coronary artery lesions. The multiplication factor for any other branch is 0.5. Female subjects were only included in the study if they were postmenopausal or surgically sterile. According to the World Health Organization (WHO) 1999 criteria [16], a person with a prior history of diabetes was classified as previously diagnosed diabetes regardless of the glucose levels, and Exclusion criteria included: unstable/uncontrolled clinically significant diseases, uncontrolled primary hypothyroidism, nephrotic syndrome, renal dysfunction, or clinically significant hematology abnormalities. Additional exclusion criteria were the presence of active liver disease or hepatic dysfunction, consumption of more than 14 alcoholic drinks per week, immunosuppressive agents, and smoked cigarettes. Patients were maintained on other medications throughout the study, with no change, including calcium channel blockers, beta blockers, glucose-lowering, diuretics and other antihypertensive therapy. Written informed consent was obtained from the patients for publication of this report and any accompanying images.

150 patients with CHD receive atorvastatin treatment at 20 mg/day for 4 weeks, and other 62 CHD patients receive simvastatin at 40 mg/day for 3 weeks. Blood samples were collected from subjects, after a 12 h overnight fast, into tubes containing 0.1% EDTA.

#### Blood samples

Fasting blood samples were collected into tubes containing EDTA before the percutaneous intervention and centrifuged at 3000 rpm for 20 min at 4°C to obtain plasma. Then it was stored at 4°C and used within 24 h for lipid and apolipoprotein analyses. An aliquot of plasma was stored at −70°C for the determination of HDL subclasses.

#### Plasma lipid and apolipoprotein analyses

Fasting plasma concentrations of TG and total cholesterol (TC), HDL-C, and low density lipoprotein cholesterol (LDL-C) along with FPG, apoA-I, apoB-100 values were measured using automated standardized by Clinical Laboratory of West China Hospital, Sichuan University.

#### HDL subclasses analyses

ApoA-I contents of plasma HDL subclasses were determined by 1-dimensional gel electrophoresis coupled with immunodetection for apoA-I which developed and modified at the Laboratory of Endocrinology and Metabolism, West China Hospital, Sichuan University.

#### Materials and equipment

The acrylamide was obtained from Serva, TRIS from CA, bisacrylamide, boric acid, ammonium persulfate, sucrose and TEMED from Merck, PVDF membrane from Millipore. The gel apparatus included the BioRAD Mini-PROTEAN 3 system, the LKB gradient maker, the LKB peristaltic pump, the BioRAD thermostatic circulator.

### Nondenaturing Polyacrylamide GGE of HDL

#### Gel casting

Polyacrylamide gradient gels were cast using a 2-chamber gradient mixer. The Casting Frame, when placed on the benchtop, evenly aligns and secures the Spacer Plate and the Short Plate together to form the gel cassette sandwich prior to casting (BioRAD Mini-PROTEAN 3 system ,1.0-mm spacers, 10-well combs). Then, 3.5 ml of the 2%acrylamide solution and 3.5 ml of a 30% acrylamide solution were poured into the 2 chambers of the gradient mixer. The acrylamide gradient was formed by allowing the gradient mixture to fill the gel casting cassette from the bottom during by hydrostatic pressure. The Casting Stand secures the Gel Cassette Assembly during gel casting. When the gradient had been formed, 1 ml of distilled water was added manually. The distilled water was added slowly with a Pipette Pasteur, taking extreme care to keep a constant flow and to avoid any stirring of the gradient. Gels were then left to polymerize at room temperature, after which they could be stored under moist conditions at 4°C refrigerator for no longer than 1 week.

#### Electrophoresis

Preelectrophoresis (30minutes at 70 V) and electrophoresis were performed by using Tris, boric acid, and Na_2_-EDTA (pH 8.4) as running buffer. Whole serum (50 μl) was mixed with 25 μl of sucrose and a total volume of 10 μl of sample was applied to each well. The 10 μl of reference proteins (HMW Calibration for Native Electrophoresis, GE Healthcare UK Ltd., Buckinghamshire, England) were run on gel. Wells closest to the edges of the gel were not used. Electrophoresis was conducted at 70 V for 30 minutes, 100 V for 6 h and 200 V for 14 h.

#### Western blotting

After electrophoresis, plasma proteins and reference proteins were electroretically transferred to PVDF membranes, stained with 0.1% ponceau S, and the position of reference protein bands labeled by pencil, and distained by diffusion, the membrane was blocked with 5% BSA and incubated with horseradish peroxidase-labeled goat anti-human apoA-I-IgG polyclonal antibody which was prepared at the apolipoprotein Research Laboratory, West China Medical Center, Sichuan University overnight at 4°C. The membrane was washed three times. The relative concentration of each subclass was calculated as the percentage of plasma apoA-I (%) according to the density of each spot (BioRAD Imaging System Flier). The relative concentration of each HDL subclasses was calculated as the percentage of total plasma apoA-I according to the density of each spot. Particle diameters of the HDL subclasses were assessed by comparing the mobility of the sample with the mobility of calibration standard using a standard curve that included thyroglobulin, ferritin, catalase, lactate dehydrogenase, and bovine serum albumin (HMW Calibration for Native Electrophoresis, GE Healthcare UK Ltd., Buckinghamshire, England). Then, the relative percent concentration of each HDL subclass was multiplied by the apoA-I concentration in the individual samples, respectively, to yield the relative concentrations of each HDL subclass of apoA-I (in mg/L). The inter-assay variation (n = 5) of the specific HDL subclasses was 9.6% (Preβ_1_-HDL), 8.5% (HDL_3c_), 6.8% (HDL_3b_), 10.3% (HDL_3a_), 9.9% (HDL_2a_), and 8.3% (HDL_2b+Preβ2-HDL_), respectively. The intra-assay variation (n = 5) of the specific HDL subclasses was 2.1% (Preβ_1_-HDL), 2.0% (HDL_3c_), 1.6% (HDL_3b_), 2.4% (HDL_3a_), 2.3% (HDL_2a_), and 1.9% (HDL_2b+Preβ2-HDL_), respectively.

#### Statistical analyses

Kolmogorov-Smirnov statistics was used to test normality of distributions of plasma lipids, lipoproteins, HDL subclasses, and clinical parameters. For analysis, non-Gaussian-distributed data were transformed using the natural logarithm to approach a Gaussian distribution. All values were presented as mean ± S.D. The significant differences between two groups were analyzed by t-test. Pearson correlation analysis was used to estimate the association between plasma parameters and the changes in HDL subclasses profile among CHD with diabetes patients. A multiple regression analysis was also preformed the relationship between changes in coronary stenosis, and plasma lipids, lipoproteins as well as the contents of HDL subclasses. Differences were considered significant at *P* < .05. All statistical analysis was performed using the statistical package SPSS Version 15.0 (SPSS Inc).

## Results

### The one-dimensional gel electrophoresis and immunodetection method

**Figure 1:**
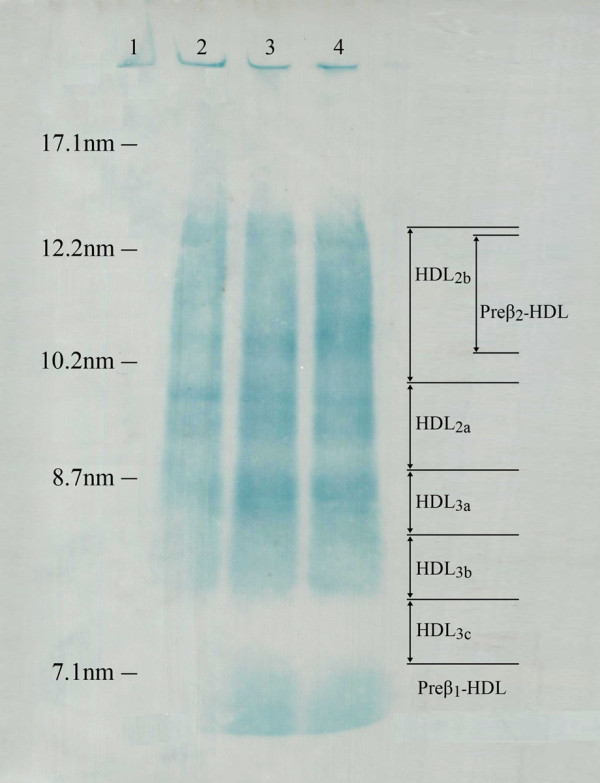
**High-density lipoprotein subclasses were separated by nondenaturing 1-dimensional gel electrophoresis and immuodetection.** The high molecular protein standards (lane 1), Normolipidemic healthy subjects (lane 2), CHD Patients (lane 3 and lane 4).

Figure 1 shows the distributions of HDL subclasses for representative CHD and normolipidemic healthy subjects by nondenaturing 1-dimensional gel electrophoresis and immunodetection method. The high molecular protein standards (lane 1), (lane 2) from healthy normolipidemic subjects, (lane 3 and lane 4) from CHD subjects. With this method, the HDL can be separated into 6 distinct subclasses, preβ_1_-HDL, HDL_3c_, HDL_3b_, HDL_3a_, HDL_2a_, and HDL_2b+ Preβ2_, according to their particles size.

**Figure 2:**
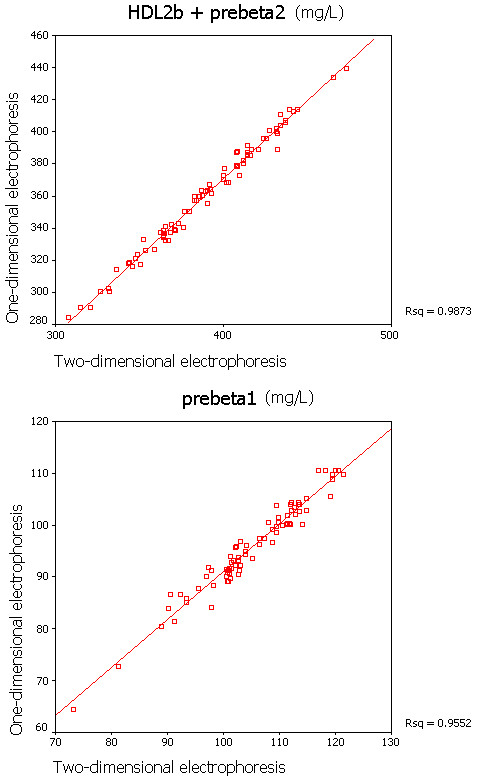
**The scatter chart of preβ**_**1**_**-HDL and HDL**_**2b+preβ2 **_**between in nondenaturing 1-dimensional and 2-dimensional gel electrophoresis coupled with immuodetection methods.**

Figure 2 presented that the liner relationship between the HDL subclasses [preβ_1_-HDL(r = .95) and HDL_2b+preβ2_(r = .98)]determined by using 1D-PAGGE associated with immunodection and those by using 2D-PAGGE associated with immunodection is stronger.

### Baseline characteristics and plasma lipid, apolipoprotein along with HDL subclasses distribution Profiles in CHD patients with and without diabetes

Plasma levels of FPG, TG, TC, LDL-C, apoB-100, and the ratios of apoB-100 to apoA-I along with waist-to-hip (WHR) as well as waist and Gensini Score were significantly higher; in contrast, plasma levels of HDL-C (−30%), apoA-I (−18%) were significantly lower in CHD patients with diabetes (n = 115) in comparison with the CHD patients without diabetes (n = 97; Table 1). The body mass index (BMI), and Hip did not differ significantly between the CHD patients with diabetes and the CHD patients without diabetes.

**Table 1:** Clinical and Biological Characteristic of CHD Patients with Diabetes and CHD Patients without Diabetes

	**CHD Patients with Diabetes (n = 115)**	**CHD Patients without Diabetes (n = 97)**
**Age(yr)**	67.1 ± 9.7	63.7 ± 8.3
**Female/Male**	45/70	53/44
**SBP(mmHg)**	135.2 ± 20.6	130.8 ± 17.7
**DBP(mmHg)**	77.6 ± 8.4	75.7 ± 7.5
**BMI(kg/m**^**2**^**)**	25.0 ± 2.6	24.3 ± 2.1
**Waist (cm)**	92.4 ± 8.6	88.1 ± 7.8^†^
**Hip (cm)**	94.2 ± 6.4	93.1 ± 5.7
**WHR**	0.98 ± 0.1	0.94 ± 0.1^†^
**Gensini Score**	53.7 ± 9.2	22.9 ± 6.3^†^
**FPG(mmol/L)**	9.0 ± 1.2	5.2 ± 0.6^‡^
**TG(mmol/L)**	2.2 ± 0.5	1.8 ± 0.6^*^
**TC(mmol/L)**	4.4 ± 1.1	3.9 ± 1.0^*^
**LDL-C(mmol/L)**	3.4 ± 1.0	2.7 ± 0.7^†^
**HDL-C(mmol/L)**	1.0 ± 0.2	1.3 ± 0.2^†^
**ApoA-I(g/L)**	1.1 ± 0.2	1.3 ± 0.3^*^
**ApoB-100(g/L)**	0.8 ± 0.2	0.6 ± 0.2^*^
**ApoB-100/A-I**	0.7 ± 0.2	0.5 ± 0.1^*^
**Preβ**_**1**_**-HDL(mg/L)**	92.0 ± 9.5	83.0 ± 7.0
**HDL**_**3c**_**(mg/L)**	83.9 ± 7.4	78.5 ± 6.9
**HDL**_**3b**_**(mg/L)**	170.2 ± 20.9	142.5 ± 18.2^*^
**HDL**_**3a**_**(mg/L)**	310.5 ± 44.7	272.5 ± 38.1^*^
**HDL**_**2a**_**(mg/L)**	190.5 ± 25.8	285.9 ± 42.2^‡^
**HDL**_**2b+Preβ2-HDL**_**(mg/L)**	311.9 ± 52.2	389.3 ± 69.5^‡^

CHD, coronary heart disease; BMI, body mass index; FPG, fasting plasma glucose; TG, triglyceride; TC, total cholesterol; LDL-C, low density lipoprotein cholesterol; HDL-C, high density lipoprotein cholesterol; ApoA-I, apolipoproteinA-I; ApoB-100, apolipoprotein B-100.

Data are presented as Mean ± S.D.

^*^P < 0.05, ^†^*P* < 0.01, ^‡^*P* < 0.001 vs CHD with diabetic patients.

What is more, plasma contents of large buoyant particles HDL_2a_ and HDL_2b+preβ2_ were significantly decreased but those of small dense HDL_3_ (HDL_3b_ and _3a_) were significantly increased in CHD patients with diabetes compared with CHD patients without diabetes.

### Correlation of plasma lipids, lipoproteins, apolipoproteins, and HDL subclasses contents for CHD patients with diabetes

As shown in Table 2, in CHD patients with diabetes, plasma FPG, TG, and LDL-C levels, Gensini Score, BMI as well as apoB-100/A-I ratio were positively correlated with HDL_3a_, and HDL_3b_ but inversely associated with HDL_2a_, and HDL_2b+preβ2_. On the contrary, HDL-C levels were negatively correlated with HDL_3a_ and positively correlated with HDL_2a_, HDL_2b+preβ2_ along with preβ_1_-HDL. The apoA-I levels were related significantly to all HDL subclasses contents.

**Table 2:** Assessment of Relationship between Plasma Lipid, Lipoprotein, Apolipoprotein and HDL Subclasses Contents in CHD Patients with Diabetes

	**Preβ**_**1**_**-HDL**	**HDL**_**3c**_	**HDL**_**3b**_	**HDL**_**3a**_	**HDL**_**2a**_	**HDL**_**2b+preβ2-HDL**_
**FPG**	.160	.108	.390^*^	.398^*^	-.570^**^	-.630^**^
**TG**	.112	.208	.243^*^	.291^*^	-.305^**^	-.369^**^
**TC**	.099	.199	.192	.210^*^	.030	.042
**LDL-C**	.140	.120	.220^*^	.150	-.252^*^	-.271^*^
**HDL-C**	.176^*^	-.140	-.136	-.174^*^	.302^**^	.358^**^
**Gensini Score**	.102	.134	.184^*^	.211^*^	-.234^**^	-.263^**^
**BMI**	.210	.231	.217	.256^*^	-.244^*^	-.270^*^
**ApoA-I**	.430^**^	.405^**^	.453^**^	.507^**^	.575^**^	.602^**^
**ApoB-100**	.116	.128	.133^*^	.101	-.120	-.123
**ApoB-100/A-I**	.240	.307^*^	.365^**^	.444^**^	-.436^**^	-.470^**^

CHD, coronary heart disease; FPG, fasting plasma glucose; TG, triglyceride; TC, total cholesterol; LDL-C, low density lipoprotein cholesterol; HDL-C, high density lipoprotein cholesterol; BMI, body mass index; ApoA-I, apolipoproteinA-I; ApoB-100, apolipoproteinB-100.

^*^P < .05, ^**^P < .01.

### Relationship of plasma lipids, lipoproteins, apolipoproteins, and HDL Subclasses contents with the gensini score by Multivariate Stepwise Regression Analyses

To obtain a better understanding of the determinants for CHD patients with diabetes, stepwise multivariate regression analyses with the Gensini Score as dependent variables, and plasma lipids, lipoproteins, apolipoproteins, and HDL subclasses as independent variables were performed in this work (Table 3). The results revealed that the Gensini Score was significantly and independently predicted by TC, apoB-100, and the large-sized HDL_2a_ along with HDL_2b+preβ2_.

**Table 3:** Correlations of HDL Subclasses Contents with Gensini Score in CHD Patients with Diabetes

			**Unstandardized Coefficients**	**Standardized Coefficients**	***t***	***P***
		***β***	**Standard error**	***β***		
**Gensini Score**	**HDL**_**2a**_	-.499	.174	-.572	−2.856	.007
	**HDL**_**2b**_	-.522	.183	-.598	−2.872	.006
	**Apo**_**B100**_	67.563	20.153	.677	2.495	.016
	**TC**	28.304	8.183	.522	3.459	.001

CHD, coronary heart disease; TC, total cholesterol; HDL-C, high density lipoprotein cholesterol; Apo_B100_, apolipoprotein_B100._

## Discussion

HDL is not a homogeneous category of lipoproteins but consists of a set of distinct subclasses of particles that vary in size, shape, density, surface charge, and composition. Nondenaturing polyacrylamide gradient gel electrophoresis (PAGGE) has been used to isolate HDL based on particle size. Human plasma HDL includes five subclasses known as HDL_2b_, HDL_2a_, HDL_3a_, HDL_3b_, and HDL_3c_ in order of decreasing particle size [17]. Other methods for measuring HDL subclasses, such as nuclear magnetic resonance (NMR) spectroscopy, which assumes the detected amplitudes of spectral signals emitted by HDL subclasses of different size. This technique determines 5 subclasses of increasing size, HDL1, 2, 3, 4, and 5 corresponds to the HDL_3c__3b__3a__2a__2b_ according to the PAGGE [18]. Agarose gel electrophoresis separates HDL into α-, and preβ-mobility HDL, according to their different charges [19]. By combining agarose gel electrophoresis with nondenaturing PAGGE (2D-PAGGE) along with immunodetection, preβ-HDL can be further separated into preβ_1_-, and preβ_2_-HDL except the α-HDL used to isolate 5 subclasses. In this work, the characterization of HDL particle size (diameter) by 1D-PAGGE along with immunodetection method which developed and modified at our Laboratory. That defined 6 subclasses: preβ_1_-HDL, HDL_3c_, HDL_3b_, HDL_3a_, HDL_2a_, and HDL_2b+preβ2_ (Figure 1). Preβ_1_-HDL has an apparent molecular mass of 60–70 kDa and a diameter of 5–6 nm [20]. Therefore, the preβ_1_-HDL can be separated easily from the other HDL subclasses components. On the other hand, because of the diameter of preβ_2_-HDL(10.2–12.37 nm) was similar to that of HDL_2b_(9.8–12.97 nm), they overlapped each other. In this context, we used to add up the preβ_2_-HDL, HDL_2b_ defined as HDL_2b+preβ2_.

Meanwhile, we have investigated the plasma HDL subclasses distribution in hyperlipidemic, and obese subjects by 2-dimensional gel electrophoresis associated with immunodection [21-23] and found that the contents of preβ_2_-HDL kept the relative constant among these subjects (about 60 mg/L). Thus, the changes in contents of HDL_2b+preβ2_ mainly reflect the alteration of HDL_2b_ contents. Moreover, relations of the preβ_1_-HDL and HDL_2b+preβ2_ by using 1D-PAGGE associated with immunodection and 2D-PAGGE associated with immunodection, respectively were explored, and the results revealed that the preβ_1_-HDL (r = .95) and HDL_2b+preβ2_ (r = .98) have a significant liner correlation between in 1D-PAGGE associated with immunodection and 2D-PAGGE associated with immunodection measuring methods (Figure 2). The other HDL subclasses contents in subjects with 1D-PAGGE associated with immunodection were similar to those in subjects with 2D-PAGGE associated with immunodection (data not shown). Based on above reasons, making use of the 1D-PAGGE associated with immunodection to classify the HDL subclasses is simple and reliable.

Our study, conducted on a the CHD patients with or without diabetes, confirmed the typical alterations of lipid profile, i.e., low concentrations of HDL-C, increased levels of TG, TC, LDL-C, and FPG in CHD patients with diabetes as compared to CHD patients without diabetes. Meanwhile, the data presented that diabetes was associated with a profound remodel of HDL subclasses toward a less atheroprotective profile, with a reduction of the larger HDL_2_ (HDL_2a_ and HDL_2b+preβ2_) subclasses, and an increase of the smaller, lipid poor HDL_3_ (HDL_3b_, and _3a_) particles.

Compared and analyzed the prevalence of coronary artery lesions according to the angiographic Gensini Score which used to reflect the extent of coronary atherosclerosis, and the data showed that in the group of CHD patients with diabetes, the Gensini Score was significant higher than that in the group of CHD patients without diabetes (53.7 vs 22.9). Regardless of in CHD patients with or without diabetes, the mean levels of plasma lipids, and lipoproteins were normal or desirable(as defined by the NCEP ATP-III guidelines) except the TG levels were border-line high and HDL-C levels were low for CHD patients with diabetes; whereas the contents of HDL_2b+preβ2_ (389.3 mg/L) were significantly lower for the CHD patients without diabetes than HDL_2b_ added preβ_2_-HDL contents (378.4 + 56.0 = 434.4 mg/L) for normolipidemic subjects [24]. Unlike this, we have previously investigated the plasma HDL subclasses distribution in hyperlipidemic, obese, primary diabetic mellitus (DM), and CHD patients [22,23,25-27]. The results showed that in these subjects, increased TG, TC, LDL-C, and decreased HDL-C accompanied with an increase of small-sized HDL particles and a reduction of large-sized HDL particles successively.

DM is characterized by dyslipidemia, including increased LDL-C levels, low HDL-C levels, and increased TG. Unlike other lipoproteins, HDL are not formed as mature lipoproteins, but appears in plasma as precursor particles. In CHD patients with diabetes, these subclasses undergo remodeling and interconversion by the addition and removal of their neutral lipids, phospholipids, and apo components due to the alteration of plasma lipids levels.

Several enzymes and proteins involved in HDL metabolism and remodeling are lecithin: cholesterol acyltransferase (LCAT), cholesterol ester transfer protein (CETP), hepatic lipase (HL) and lipoprotein lipase(LPL) and phospholipids transfer protein (PLTP), the ATP-binding cassette transporter A1(ABCA1) and G1(ABCG1). LCAT transfers the sn-2-acyl group of lecithin to cholesterol, therefore results in the generation of cholesterol esters, which are retained in the core of HDL particles, leading to the conversion of discoidal nascent HDL into spherical particles. CETP action results in transfer of CE from HDL towards triglyceride-rich lipoproteins (TRLs), and reciprocal transfer of TGs from TRLs to HDL. Elevated TGs increase the CE transfer process out of HDL, resulting in CE depletion and TG enrichment of HDL and subsequent increased HDL catabolism leads to the formation of small HDL particles [28]. LPL is the rate-limiting enzyme for the hydrolysis of the TG core of TRLs, chylomicrons (CM) and VLDL, when catabolized by LPL, CM and VLDL release PL, apoA-I, cholesterol, TG, and apoCs, subsequent binding of these products to HDL_3_ results in the formation of HDL_2_ particles [29]. ABCAI is responsible for cholesterol and PL efflux towards lipid-poor or lipid-free apoA-I [30]; ABCG1 mediates cholesterol and PL efflux to mature HDL [31].

Most studies have established the action of above enzymes and protein factors associated with the increase of plasma TG, LDL-C, and decrease of plasma HDL-C levels. Hence, based on the changed in plasma lipids which leading to altered HDL subclass and particle size remodel in CHD patients with diabetes [32,33].

Meanwhile, plasma LCAT and LPL activity has been reported to be decreased in IR and T_2_DM subjects [34]. A decreased post-heparin plasma LPL/HL ratio is a determinant of low HDL_2_ cholesterol in IR. At the same time, Mauldin et al [35] could demonstrate that macrophage ABCG1 expression and function are decreased in mouse models of T_2_DM and ABCA1 gene expression is severely decreased in the liver and pertitoneal macrophages of diabetic mice compared with euglycemic control mice [36]. In addition, PLTP activity in plasma is elevated in IR and T_2_DM in association with high plasma TG and obesity [37].

Compared with the CHD patients without diabetes, in CHD patients with diabetes, the alteration of plasma lipids levels and activities of enzymes involved in HDL particles metabolism and the receptor mediated uptake or efflux of free cholesterol(FC)/cholesterol esters(CE) towards/from HDL is modified result in altered HDL subclass and particle size.

The changes in HDL subclasses might correlate with the WHR. The present data displayed that WHR is higher significantly in CHD patients with diabetes (0.98), as compared to CHD patients without diabetes (0.94). Some studies demonstrated a negative relationship between WHR and HDL_2_, independent of TG and other subclasses [38]. This concords with studies by Terry et al [39] concerning HDL_2_, although the latter did not correct for TG and Walton et al [40] where TG were taken into account.

Type 2 diabetes is largely considered as a CHD “equivalent” risk category [41] and the differences in HDL subpopulations profile noted in diabetic women resembled, both qualitatively and quantitatively, those reported for male subjects with a previous CHD event [42]. Taken together these findings suggest that early alterations in HDL subpopulations profile may contribute to CHD risk in diabetic patients.

It was demonstrated that atorvastatin was more effective to treat lipid abnormalities and to modify the HDL subclasses profile than simvastatin, by increasing the concentration of the large, cholesterol rich, HDL_2_ and decreasing the concentration of the small, TG-rich, HDL_3_ in CHD patients [43]. In our study, nevertheless statins therapy could be effective in normalizing the plasma lipids phenotype, the HDL particle size tend to small in CHD patients with or without diabetes, particularly in CHD patients with diabetes. That implicated that the distribution of HDL subclasses modification lagged behind the improvement of plasma lipids levels. For the possible reason, on one side might involve in the conversion process of HDL subclasses. On the other side, it is well known that the treatment of statins had significantly effect in modulating the atherogenic lipids; but the impact of it on plasma lipoproteins metabolism is unclear, and we speculated that the compositions of plasma lipoproteins change could play a function in these, and need to further study. Meanwhile, the Pearson correlation and multivariate stepwise regression analysis supported that the Gensini Score were associated independently with HDL subclasses. Under this condition, our findings implication that the distribution of HDL subclasses abnormalities might be an important potential factor involved in the increased CHD risk associated with diabetic patients and arteriographic progression.

In present study, we mainly discussed the HDL subclass profiles and alterations of particle size in CHD patients with diabetes or without diabetes. The limitation of the present work is that no baseline measurements of lipids prior to beginning statin therapy, and potential mechanisms underlying the processing and/or distribution difference in HDL subclasses particles should be discussed in next study.

## Conclusions

To summarize, the abnormality of HDL subpopulations distribution and particle size may contribute to CHD risk in diabetic patients. The HDL subclasses distribution may help in severity of coronary artery and risk stratification, especially in CHD patients with therapeutic LDL, TG and HDL levels.

## Abbreviations

HDL: High-density lipoprotein; CHD: Coronary heart disease; LDL-C: Low density lipoprotein; TC: Total cholesterol; FPG: Fasting plasma glucose; TG: Triglyceride; DM: Diabetes mellitus; CAD: Coronary artery disease; CV: Cardiovascular; QCA: Quantitative coronary angiography; LMS: Left main stem; LAD: Left anterior descending artery; CX: Proximal circumflex artery; WHO: World Health Organization; WHR: Waist to hip; BMI: Body mass index; NMR: Nuclear magnetic resonance; NCEP: National Cholesterol Education Program; IFG: Impaired fasting glucose.

## Competing interests

The authors declare that they have no competing interests.

## Authors’ contributions

LT participated in the design of study and paper preparation together with editing. SYL helped to review the manuscript. CWL collected and performed data. YHL participated in drafted the manuscript. YCC performed the data acquisition and analysis. ZZ conceived of the study. MDF participated in manuscript reviewing and drafting. All authors read and approved the final manuscript.
